# Suppression of Fusarium wilt of cucumber by ammonia gas fumigation via reduction of *Fusarium* population in the field

**DOI:** 10.1038/srep43103

**Published:** 2017-02-23

**Authors:** Jun Zhao, Zhong Mei, Xu Zhang, Chao Xue, Chenzhi Zhang, Tengfei Ma, Shusheng Zhang

**Affiliations:** 1 School of Agriculture and Biological Engineering, Jinhua Polytechnic, Jinhua, 321007, Zhejiang, China; 2 Jiangsu Provincial Key Lab for Solid Organic Waste Utilization, Nanjing Agricultural University, 210095, China

## Abstract

Cucumber plants subjected to consecutive monoculture for 9 years were found to suffer from severe Fusarium wilt disease, caused by the soil-borne fungus *Fusarium oxysporum* f. sp. *Cucumerinum* J. H. Owen. In the present study, greenhouse experiments were performed to evaluate the influence of ammonia gas fumigation on Fusarium wilt suppression, fungal abundance and fungal community composition. Results showed that ammonia gas fumigation remarkably reduced disease incidence from 80% to 27%, resulting in a four-fold increase in yield, compared to the control. Total fungal abundance declined dramatically after fumigation and reached the lowest level at day 32, at 243 times lower than the control. Moreover, fumigation significantly increased soil fungal diversity, though it also decreased considerably coinciding with cucumber growth. Fumigation also significantly altered soil fungal community composition, relative to the control. *Fusarium* was strongly inhibited by fumigation in both relative abundance (3.8 times lower) and targeted quantification (a decrease of 167 fold). Collectively, the application of ammonia gas fumigation to control Fusarium wilt of cucumber resulted in a re-assembly of the fungal community to resemble that of a non-disease conducive consortium. Additional strategies, such as bioorganic fertilizer application, may still be required to develop sustainable disease suppression following fumigation.

Cucumber (*Cucumis sativus* L.) is an important global vegetable crop whose production can be severely hindered by infection with *Fusarium oxysporum* f. sp. *Cucumerinum* J. H. Owen. This pathogen causes Fusarium wilt disease with symptoms that include necrotic lesions and vascular and root wilt that ultimately lead to plant death^1^. Fusarium wilt has been reported to be one of the most destructive diseases in cucumber production in China and worldwide^2^,^3^. Once a soil is infected, it is virtually impossible to remove the pathogen, with survival up to 20 years. This disease can occur throughout all growth stages of cucumber and the severity is less at adult stages^4^.

To control Fusarium wilt of cucumber, our previous studies revealed that the application of bioorganic fertilizer composed of a combination of biocontrol agents and compost decreased disease incidence with a concomitant increase in yield^5^,^6^,^7^. However, there appears to be a limit to this biocontrol. For example, when the disease incidence rate is over 80%, bioorganic fertilizer may not suppress this disease. Soil fumigation is required in these instances to control Fusarium wilt of cucumber. The most effective fumigant is methyl bromide, which can effectively control soil-borne disease by killing the causative fungal pathogens. However, the use of methyl bromide has been banned under the Montreal protocol since 2004 due to its ozone-depleting characteristics^8^,^9^. As such, there is an urgent need for the development of a novel, more environmentally friendly fumigant.

Ammonium bicarbonate as an alternative fumigant was evaluated for efficacy against soil-borne diseases including southern stem rot, Sclerotinia blight of peanut^10^ and Fusarium wilt of cucurbits^11^. One of the key mechanisms is the production of ammonia after application that has been used as food product antimicrobial agent for over a century. As early as 1895, ammonia was introduced to preserve beef tea. Currently, anhydrous ammonia is used commercially at the post-harvest stage to reduce or eliminate microbial contamination of corn, citrus, alfalfa seeds, mung beans, and poultry production^12^,^13^,^14^,^15^,^16^. In the hydrous form, ammonium hydroxide has also been widely applied as N fertilizer when urea was not widely available^17^.

In this study, anhydrous ammonium (ammonium gas) was applied in an alternative soil fumigation approach to control Fusarium wilt of cucumber. Greenhouse experiments were performed to evaluate the effects of anhydrous ammonium on disease control efficacy, cucumber yield, soil fungal population, fungal community composition and diversity using a high-throughput sequencing approach coupled with quantitative PCR.

## Results

### Field experiment

**Figure 1:**
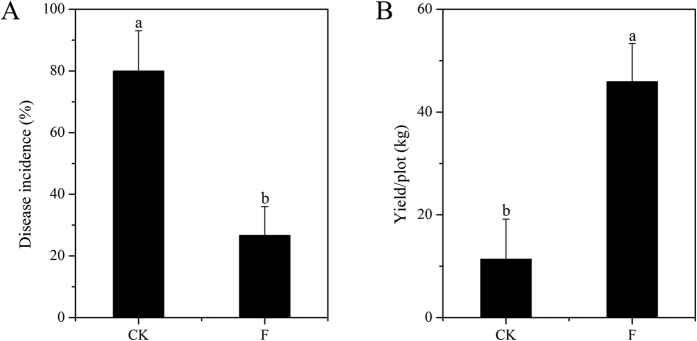
Disease incidence (**A**) and cucumber yield (**B**) of the control (CK) and fumigation (F) treatments. The significance of the difference was determined by two-tailed unpaired t-tests. Bars with different letters represent significant difference at p < 0.05.

Disease incidence in the control (CK) and fumigation (F) treatments showed that wilt disease was extremely severe with an average incidence rate of 80% in the CK treatment while the F treatment significantly (p < 0.05) decreased disease incidence to 26.7%, resulting in a control efficacy of 66.7% (Fig. 1A). In addition, cucumber yield was increased significantly (p < 0.05) in the F treatment (45.95 kg/plot), compared to the CK treatment (11.38 kg/plot) (Fig. 1B).

### Fungal quantification

**Figure 2:**
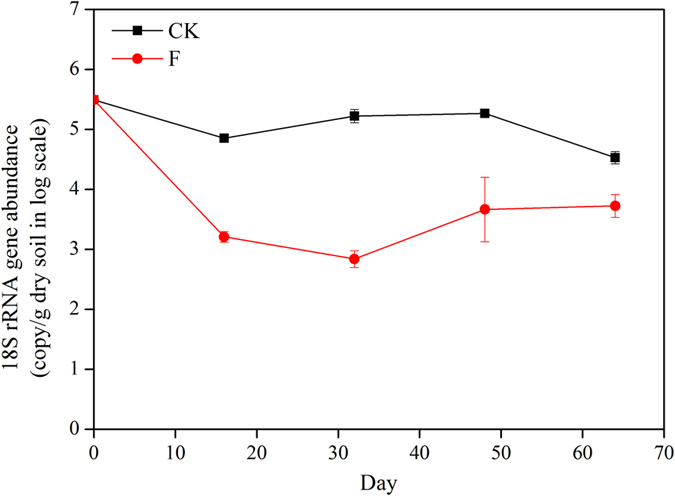
Abundance of the total fungal community in the control (CK) and fumigation (F) treatments based on quantification of 18S rRNA genes.

Overall, the fungal population in the CK treatment was relatively stable (ranging from 3.37 × 10^4^ to 3.1 × 10^5^) throughout the entire growing season, with a slight increase corresponding to plant growth and a moderate decrease after cucumber harvest (Fig. 2). Fungal community abundance declined dramatically in the F treatment, reaching its lowest level at day 32 at 243 times lower than the CK treatment (p < 0.05). After day 32, fungal abundance resurged, followed by stabilization for the remainder of the growing season at an abundance 6 times lower than the CK treatment after cucumber harvest (p < 0.05). Overall, fungal abundance was significantly (p < 0.05) lower in the F treatment over the course of the experiment, demonstrating that ammonia gas fumigation was able to effectively suppress the fungal population during the entire cucumber growing season. Fungal abundance was significantly positively correlated with disease incidence (r = 0.90, p < 0.001) and negatively correlated with cucumber yield (r = −0.90, p < 0.001).

### Fungal richness and diversity

Fumigation significantly (p < 0.05) increased the Shannon diversity of the fungal community, yet there was no considerable influence on Chao1 richness (Table 1). In both treatments, diversity initially increased from time 0 (CK0), followed by a decrease from seedling transplantation to cucumber harvest. In the CK treatment, diversity was significantly (p < 0.05) lower after harvest (CK4), compared to the CK0. In contrast, fungal diversity in the F treatment was higher (p < 0.05) at the end of the growing season (F4), compared to the CK0. In addition, fungal richness decreased from CK0 through cucumber harvest in both treatments.

### Fungal community structure

**Figure 3:**
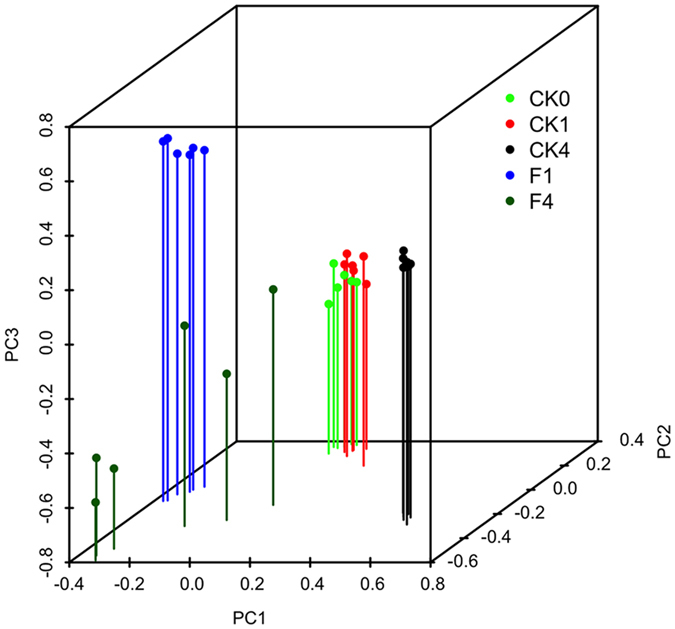
Principle component analysis (PCA) based on Bray-Curtis dissimilarities between all sample sets. F and CK are fumigation and control treatments, respectively. “0”, “1”, and “4” represent before fumigation, after seedling transplantation and harvest, respectively.

**Figure 4:**
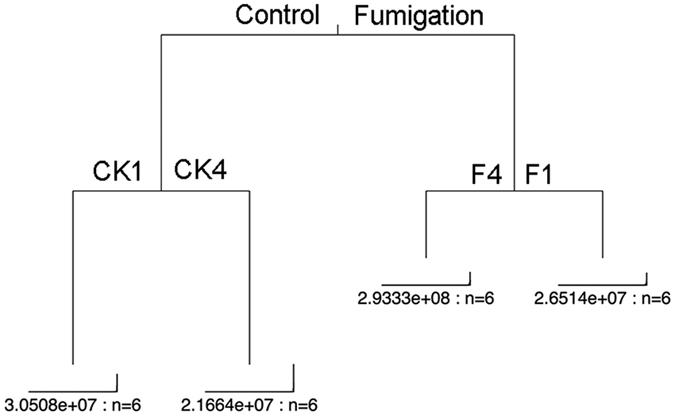
Multiple regression tree (MRT) analysis of time × crop growth stage and treatment effects on fungal composition. F and CK represent fumigation and control treatments, respectively.

Soil fungal community composition was influenced by the interaction of time × crop growth stage and fumigation (Fig. 3). Fumigation resulted in distinct soil fungal communities, compared to both the CK treatment and the initial soil condition (CK0). High replicate dispersion among the harvest samples in the F treatment was also identified (Fig. 3). Fungal community composition within both the seedling transplantation (CK1) and initial soil (CK0) samples were similar with significant changes occurring at the harvest stage (CK4). Furthermore, multiple regression tree analysis confirmed that fumigation overcame the effect of both crop growth stage and time on soil fungal community composition (Fig. 4).

### Fungal community composition

**Figure 5:**
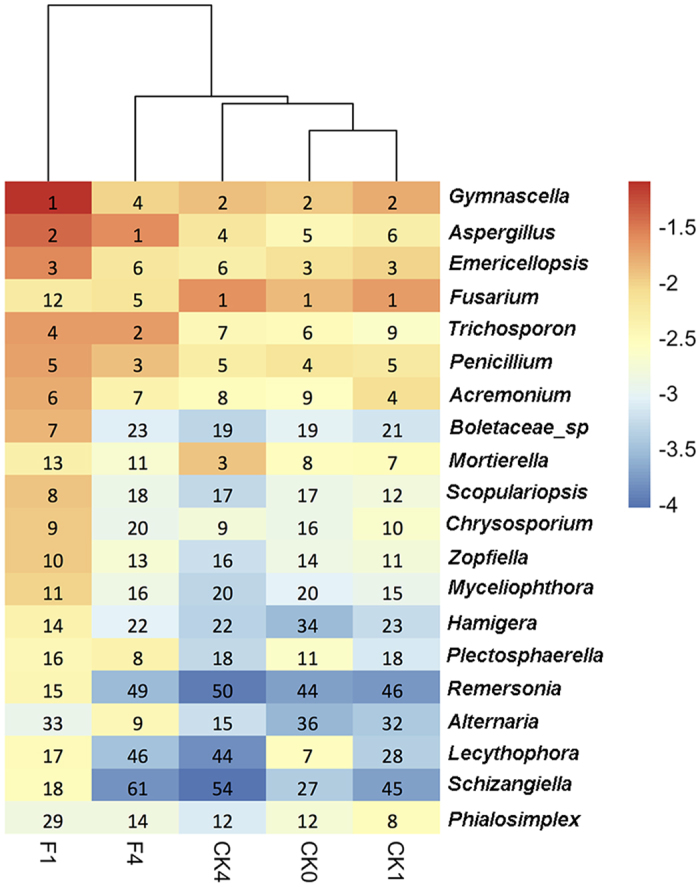
Heatmap displaying the relative abundances of top 20 fungal genera for all treatments. The key from blue to red represents the least abundant to most abundant. The numbers represent the rank of the fungal genera in each treatment sorted from the most abundant to the least abundant genus.

The relative abundance of the top 20 fungal genera varied principally between the CK and F treatments (Fig. 5). Interestingly, *Fusarium* was the most abundant fungal genus in the CK treatment as well as the initial soil (CK0), whereas the relative abundance was notably reduced in the F treatment. This suggests that *Fusarium* was sensitive to fumigation and suppressed for the entire crop season. Relative abundances of the majority of the top 20 fungal genera were considerably increased after fumigation (F1). However, a proportion of these genera decreased in abundance during plant growth and became consistent with the control at the harvest stage, indicating that the fungal community composition tended to gradually recover from fumigation after one crop season. Additionally, the relative abundances of *Emericellopsis, Penicillium* and *Phialosimplex* were similar in the CK and F treatments, indicating a lack of sensitivity to ammonia gas fumigation. Similarity percentage (SIMPER) analyses further revealed that *Gymnascella, Aspergillus, Trichosporon, Fusarium*, and *Penicillium* were the top 5 most abundant fungal genera contributing to the dissimilarity between the CK and F treatments. Overall, the top 10 contributing genera comprised 70% of the total Bray-Curtis dissimilarity with 7.4% and 15.7% of the sequences in CK and F treatments, respectively (Table S1).

**Figure 6:**
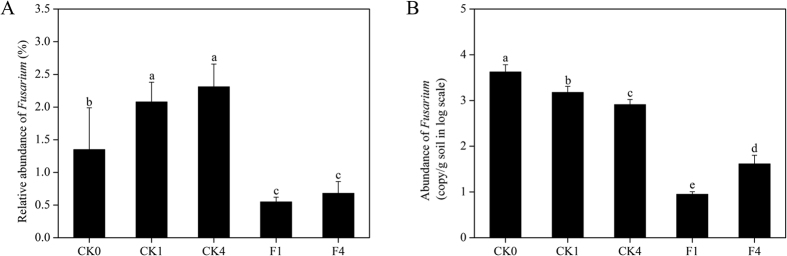
Abundance of Fusarium in F (fumigation) and CK (control) treatments. (A) Relative abundance of Fusarium based on high throughput sequencing data. (B) The copy number of Fusarium was calculated according to *Fusarium* abundance × fungal copy number. The significance of the difference was determined by one-way ANOVA (n = 6). Bars shared the same character represent a lack of significant difference (p > 0.05).

The relative abundance of *Fusarium* dropped significantly (p < 0.05) in the F treatment (F1, F4), but increased considerably (p < 0.05) after planting in the CK treatment (CK1, CK4), compared to the initial soil (CK0) (Fig. 6A). In addition, *Fusarium* abundance increased (p > 0.05) during plant growth in both treatments, suggesting that cucumber root exudates may have a stimulatory effect on *Fusarium* abundance. Pearson correlation revealed that *Fusarium* abundance was significantly correlated with disease incidence (r = 0.85, p < 0.01) and cucumber yield (r = −0.87, p < 0.01).

*Fusarium* copy number was calculated according to the *Fusarium* relative abundance × fungal population for each sample (Fig. 6B). Copy numbers dropped dramatically (p < 0.05) after fumigation (F1), at levels 167 times lower than the control (CK1), coupled with a significant increase (p < 0.05) after cucumber harvest (F4). At the end of F treatment growing season (F4) *Fusarium* abundance remained 102 times lower than the initial soil (CK0) and 20 times lower than the control (CK4). Surprisingly, the *Fusarium* copy number declined significantly (p < 0.05) not only after planting (CK1, CK4 vs. CK0), but also in concert with cucumber growth (CK1 vs. CK4). This suggests that the reduction in fungal abundance from the initial soil condition (CK0) to seedling transplantation (CK1) and then to cucumber harvest (CK4) may drive this result. *Fusarium* abundance was positively correlated with disease incidence (r = 0.91, p < 0.01) and negatively correlated with cucumber yield (r = −0.91, p < 0.01).

## Discussion

### Field performance after fumigation

Continuous monoculture often causes soil pathogen accumulation, thereby leading to severe soil-borne disease that ultimately drives a non-plant-preferred soil microbial community^18^. In this study, after 9 years of consecutive cucumber monoculture, Fusarium wilt disease of cucumber was extremely severe, with a disease incidence rate approaching 80%. In order to test an alternative fumigation strategy on disease incidence and soil fungal community composition, ammonia gas was applied under plastic film cover during the pre-plant stage in the field to control this soil-borne disease. This resulted in a control efficacy of 66.7% with a four-fold yield increase. This efficacy exceeds that identified by other studies on Fusarium wilt disease control on cucumber^7^, banana^18^ and watermelon^19^ using other control strategies.

### Fungal community influenced by growth stages

Fungal abundances slightly decreased after cucumber seedling transplantation (Fig. 2), likely due to the initial tillage that tends to destroy fungal hyphae, resulting in an overall negative impact on abundance^20^. Overall, fungal community abundance increased in the early growth stages, followed by a decrease that approached the CK harvest stage abundance. During plant growth, cucumber roots release an increasing quantity of root exudates^21^, which may provide sufficient stimulation of the fungal community to support growth. However, in the CK treatment, cucumber senescence began at the blooming stage (~40 days after seedling transplantation), possibly imposing resource limitations on the fungal community. Conversely, fungal abundance increased and then became steady after blooming stage in the F treatment, further supporting that healthy cucumber plants are the determinant factor in supporting fungal population proliferation.

Tillage impacted soil fungal community composition though the influence was minimal compared to that imposed by growth stage (CK4 vs. CK0), indicating that growth stage was a major deterministic factor influencing soil fungal community composition. Broeckling, *et al*.^22^ demonstrated that soil fungal community composition was largely driven by root exudates. Moreover, plants secrete distinct root exudates both qualitatively and quantitatively at different growth stages. This exerts a selection effect, resulting in community variations that coincide with plant growth^21^,^23^,^24^, similar to that observed in this study. Previous studies also suggested that seasonal changes such as temperature, moisture content and nutrient levels, among others, may impact soil microbial community composition^25^,^26^,^27^. As such, it is reasonable to assume that these seasonal changes may have also partially impacted our study findings.

Cucumber growth coincided with gradual increases in the average relative abundances of *Fusarium*, though these increases were insignificant due to larger replicate variations. However, the calculated *Fusarium* population decreased after the growing season in the CK treatment, inconsistent with numerous previous studies that showed an increase in *Fusarium* abundance after the growing season^2^,^6^,^18^. This may be attributed to the decrease in the total fungal population at CK1 and CK4, characterized by the severe disease incidence in this treatment.

### Fungal community influenced by fumigation

In the field, fumigation not only resulted in an immediate and dramatic decrease in fungal abundance, but also incurred a legacy effect by a further reduction in abundance to the lowest level 32 days after fumigation, with abundances 243 times lower than the control (Fig. 2). After 32 days, the fungal community began recovery and abundances increased gradually, together indicating that ammonia gas fumigation effectively suppressed the abundance of soil fungi for at least one month. The root system of cucumber seedlings recruited beneficial microorganisms in the rhizosphere during this time that may prevent subsequent plant pathogen infection through resource and niche competition^28^,^29^,^30^,^31^.

Surprisingly, fumigation significantly increased soil fungal diversity, inconsistent with a previous study finding^32^ that chloroform fumigation reduced microbial diversity in a soil incubation experiment. However, in the present study, soil fungal diversity was already suppressed after 9 years under consecutive cucumber monoculture. After fumigation, the re-structured fungal community was then under the influence of root exudates, which possibly impacted the restoration of fungal diversity after planting. In contrast to fungal diversity, there was no dramatic impact on fungal richness after fumigation, which then provided a basis for the recovery of the fungal community. Moreover, fumigation dramatically altered soil fungal community composition and likely induced a re-assembly of a non-disease conducive community from an originally *Fusarium* dominated community, which may play an important role in disease control throughout the growing season^33^,^34^,^35^.

Pathogen suppression is likely a key factor in controlling soil-borne disease^36^,^37^. Ammonia gas application not only reduced the overall fungal abundance but also reduced the abundance of *Fusarium*. This finding is in agreement with a previous study on cucurbits that found that ammonium bicarbonate fumigant application significantly decreased the abundance of the target pathogen through destruction of *Fusarium* mycelia^11^. However, *Fusarium* abundance increased during cucumber growth in this study, likely due to positive stimulation by specific root exudes such as cinnamic acid and phenolic acids^2^,^27^,^38^. In addition, the *Fusarium* population remained 20 times lower than the control (CK4) and 102 times lower than the initial soil condition (CK0) at cucumber harvest, indicating that the fumigation effectively suppressed *Fusarium* abundance and Fusarium wilt of cucumber for at least one crop season. Not surprisingly, different fungal genera responded dissimilarly to ammonia gas fumigation (Fig. 5), in accordance with previous results reported by Brenneman, *et al*.^10^. Particularly, ammonium bicarbonate resulted in different level of inhibition on the mycelial growth of *Sclerotinia minor, Sclerotium rolfsii*, and *Rhizoctonia solani* AG-4. Particularly, it appears that the *Fusarium* was more sensitive to ammonia gas than other fungal groups (Fig. 5), thus we propose ammonia gas fumigation could be applied in *Fusarium* spp. caused plant disease control. Moreover, future management of Fusarium wilt might need combination of multiple strategies such as soil fumigation followed by bioorganic fertilizer application to develop sustainable disease suppression.

## Materials and Methods

### Field experiment description

The experimental site is located in Jinhua, Zhejiang Province, China. Cucumbers were continuously planted for 9 years ultimately resulting in extremely severe Fusarium wilt disease, with an incidence approaching 80% in the last cropping season. To test ammonia gas fumigation on Fusarium wilt suppression, two field treatments were utilized: (1) control leaving the field untreated (CK) and (2) ammonia gas fumigation (F).

Field soil within the greenhouse was fumigated using ammonia gas. Briefly, soil was covered with plastic film and ammonia gas was applied for 3 days at a final concentration of 3500 ppm. After another 3 days, fumigation was concluded and the cover was removed. Field was tilled and cucumber seedlings (Jinyou No. 1) with three to four true leaves were transplanted to the field 5 days later. Each treatment had 6 replicated plots (~7 m^2^) and each plot had 20 cucumber plants.

The disease incidence (DI) and cucumber yield were calculated immediately after harvest. DI was evaluated by plotting the percentage of infected plants divided by the total number of plants. The control efficacy was obtained according to the equation: S = (A–B)/A, where A is the DI of the CK treatment and B is the DI of the F treatment. Plot yield was calculated as the cumulative weight of cucumbers harvested in each plot.

### Soil sampling and DNA extraction

Soil samples were collected every 16 days (numbered from 0 to 4) from the beginning of fumigation (CK0 collected on July 11, 2013) to harvest (CK4 and F4 collected on September 12, 2013 in CK and F treatments, respectively). In each plot, 5 soil cores (0–20 cm) were collected and mixed as one soil sample. DNA extraction using the Power soil DNA Extraction kit (MOBIO Laboratories, Carlsbad, CA, USA) was conducted following the manufacturer’s instructions.

### Real-time PCR assay and Miseq sequencing

Real-time PCR was performed on an ABI 7500 Cycle (Applied Biosystems, Germany) to determine the abundance of fungal 18S rRNA genes using the primer set NS1 (5′-GTAGTCATATGCTTGTCTC-3′) and Fung (5′-ATTCCCCGTTACCCGTTG-3′)^2^. The reaction mixture contained 10 μl of *Premix Ex Taq*^*TM*^ (2× ) (Takara), 0.4 μl of ROX Reference Dye II (50×), 0.2 mM of each primer, and 2 μl of template DNA with a final volume of 20 μl. Thermocycling conditions were set as follows: 30 s at 95 °C, followed by 40 cycles of 5 s at 95 °C and 34 s at 60 °C. An external calibration curve was generated according to Zhao, *et al*.^39^ with an amplification efficiency of 102.3% and an R^2^ of 0.999. Amplification specificity was verified by melting-curve analysis and agarose gel electrophoresis. Copy numbers were log_10_-transformed to normalize values prior to statistical analysis.

In order to characterize fungal community composition in response to fumigation, DNA isolated from soil samples collected before fumigation (CK0), after seedling transplantation (CK1 and F1), and cucumber harvest (CK4 and F4) were selected for Miseq sequencing. The fungal ITS region was amplified using the primer set miseq-ITS1f (5′-adapter-barcode-CTTGGTCATTTAGAGGAAGTAA-3′) and miseq-ITS2 (5′-adapter-barcode-GCTGCGTTCTTCATCGATGC-3′)^40^. After PCR amplification, the PCR products were purified using the SequalPrep Normalization Plate (96) Kit. Purified amplicons were then pooled in equimolar concentrations and subjected to sequencing at Bion Biotech Co., Ltd. (Nanjing, China). All sequences were deposited in the NCBI Sequence Read Archive (SRA) database (Accession number: SRR3064493).

### Sequence processing

Sequences were processed using the RDPipeline initial process to assemble paired-end reads and to remove low quality (Q score < 25) and short reads ( <220 bp)^41^. Chimera reads were removed through UCHIME^42^ running in de novo mode. Singletons were filtered and all samples were rarefied to the same number of reads as the sample with the lowest reads (32,427 reads per sample). Singletons were defined where only one sequence was present among all samples.

### Community data analysis

Fungal alpha-diversity (Chao1 estimator and Shannon diversity) indices were calculated based on the rarefied OTU table. Principle component analysis (PCA) was performed to examine beta-diversities (Bray-Curtis distances) between individual samples. Permutational multivariate analyses of variance (PERMANOVA)^43^ was used to determine the significance of community composition differences between treatments. Multiple regression tree (MRT) analysis was conducted to evaluate the effects of F treatment and time × crop growth stage on the whole soil fungal community^44^. Permutational analysis of multivariate dispersions (PERMDISP) was applied to evaluate the significant differences in replicate dispersion among samples^45^. Similarity percentage (SIMPER) analysis was performed to elucidate the indicator genera to the overall Bray-Curtis distances between treatments^46^.

Statistical analysis was performed using IBM SPSS statistics Version 20 (IBM Corporation, New York, United States), and complementary calculations were carried out in Microsoft Excel 2003. Two-tailed unpaired t-tests were used to compare the disease incidence and cucumber yield between CK and F treatments. Pearson correlations were performed to examine the relationships between fungal population, *Fusarium* abundance and *Fusarium* population and disease incidence as well as cucumber yield. For each variable measured in the soil, the data were analyzed by one-way ANOVA using Tukey’s HSD test (p < 0.05).

## Additional Information

**How to cite this article:** Zhao, J. *et al*. Suppression of Fusarium wilt of cucumber by ammonia gas fumigation via reduction of *Fusarium* population in the field. *Sci. Rep.* **7**, 43103; doi: 10.1038/srep43103 (2017).

**Publisher's note:** Springer Nature remains neutral with regard to jurisdictional claims in published maps and institutional affiliations.

## Supplementary Material

Supplementary Table S1

## Figures and Tables

**Table 1:** Influence of fumigation on soil fungal diversity and richness.

Treatment^a^	Chao1	Shannon
CK0	509 ± 27 a	2.55 ± 0.08 c
CK1	478 ± 22 a	2.69 ± 0.12 c
CK4	460 ± 75 a	2.05 ± 0.06 d
F1	486 ± 29 a	3.69 ± 0.19 a
F4	462 ± 20 a	3.03 ± 0.40 b

Values (mean ± SD, n = 6) within the same column followed by different letters are significantly different at p < 0.05 according to the Tukey’s HSD tests.

^a^F and CK are fumigation and control treatments, respectively. “0”, “1”, and “4” represent before fumigation, after seedling transplantation and harvest, respectively.
