# Supplementary material for: Suppression of Fusarium wilt of cucumber by ammonia gas fumigation via reduction of Fusarium population in the field
Source: Sci Rep. 2017 Feb 23;7:43103. doi: 10.1038/srep43103 (PMC5322401; doi:10.1038/srep43103)
Supplement: Supplementary Table S1 [file srep43103-s1.doc]

**Suppression of Fusarium wilt of cucumber by ammonia gas fumigation via reduction of *Fusarium* population in the field**

Jun Zhao^1,2†^, Zhong Mei^1†^, Xu Zhang^2^, Chao Xue^2^, Chenzhi Zhang^2^, Tengfei Ma^2^, Shusheng Zhang^1^*

^1^School of Agriculture and Biological Engineering, Jinhua Polytechnic, Jinhua, 321007, Zhejiang, China

^2^ Jiangsu Provincial Key Lab for Solid Organic Waste Utilization, Nanjing Agricultural University, 210095, China.

^†^These authors contributed equally to this paper

***Corresponding author**: Shusheng Zhang; Tel: (86)-0579-82230085; Fax: (86)-0579-82230086; E-mail: [sszhang62@163.com](mailto:sszhang62@163.com)

**Table S1** Similarity percentage (SIMPER) analysis showing the top contributing fungal genera to the differences between control (CK) and fumigation (F) treatments

| Genus | CK | F | Cumulative dissimilarity |
| --- | --- | --- | --- |
| *Gymnascella* | 1.51% | 4.33% | 15.80% |
| *Aspergillus* | 0.58% | 3.05% | 29.20% |
| *Trichosporon* | 0.29% | 1.89% | 38.66% |
| *Fusarium* | 2.14% | 0.78% | 47.81% |
| *Penicillium* | 0.60% | 1.51% | 53.17% |
| *Emericellopsis* | 0.73% | 1.55% | 58.34% |
| *Mortierella* | 0.72% | 0.37% | 61.76% |
| *Acremonium* | 0.56% | 0.97% | 65.17% |
| *Scopulariopsis* | 0.11% | 0.60% | 67.63% |
| *Zopfiella* | 0.12% | 0.60% | 70.02% |
